# A New Monitoring Strategy of Large Micro-, Meso- and Macro-Litter: A Case Study on Sandy Beaches of Baltic Lagoons and Estuaries

**DOI:** 10.1007/s00267-022-01755-z

**Published:** 2022-11-25

**Authors:** Greta Gyraite, Mirco Haseler, Arūnas Balčiūnas, Viktorija Sabaliauskaitė, Georg Martin, Greta Reisalu, Gerald Schernewski

**Affiliations:** 1 Leibniz Institute for Baltic Sea Research, Seestraße 15, 18119 Rostock, Germany; 2 Marine Research Institute, Klaipeda University, Universiteto al. 17, 92295 Klaipeda, Lithuania; 3 Estonian Marine Institute, University of Tartu, Mäealuse 14, 12618 Tartu, Estonia

**Keywords:** Litter pollution, Monitoring, Inner-coastal waters, Baltic Sea

## Abstract

Coastal lagoons and estuaries are hot spots to accumulate river basin-related plastic leakage. However, no official methodology exists to investigate their relatively short, rich in organic matter beaches, and the knowledge of pollution of lagoons is scarce worldwide. This study aimed to develop a methodology suitable for large micro (2–5 mm), meso (5–25 mm), and macro-litter (>25 mm) monitoring at sandy inner-coastal waters that would provide comparable results to the intensively used OSPAR 100 m method. The method proposed in this study is based on two 40 m^2^ rectangular polygons placed on the tidal accumulation zone for macro-litter enumeration and two 1 m^2^ squares for micro- and meso-litter. This method has been applied to 23 beaches from three inner-coastal waters of the Baltic Sea. This study shows that the litter densities between lagoons and bays differ and depend on the river output intensity and the retention capacity. The “Construction material”, “Plastic pieces 2–5 mm”, and “Plastic pieces 5–25 mm” were among this study’s top ten most common litter items. Experts allocated these items to the “Land based industry and trade” source, which indicates that lagoons and bays through the connection of the major rivers could be a potential sink of land-based litter. An evident strength of the methodology established is the capability to determine litter of all sizes, low-cost and time-efficiency, implementable for volunteer-based monitoring; provides comparable results to the most commonly used methods for investigating litter pollution on coastal beaches.

## Introduction

Marine litter is found all around the world in all marine habitats (Pham et al. 2014), causing damage to wildlife (UNEP 2015), leading to economic losses and safety risks to people’s life (HELCOM 2015). The majority of marine litter consists of plastic (Reisser et al. 2013), making it one of the significant environmental issues of our planet (Fallati et al. 2019) at our time (Urban-Malinga et al. 2020). The number of species negatively affected by plastic has increased to more than 500 among all wildlife groups (Kühn et al. 2015). Plastic occurs in the deep sea (Van Cauwenberghe et al. 2013), in the Antarctic (Lacerda et al. 2019), in the open ocean (Eriksen et al. 2014), while the pollution on coastlines such as salt marshes, estuaries, mangroves, and beaches (UNEP 2016) is one of the most obvious signs of it (JRC 2011).

The Marine Strategy Framework Directive (MSFD) was adopted to protect the marine environment in 2008. MSFD is aiming to reach Good Environmental Status (GES) across the European Union (EU) by 2020 through the use of 11 descriptors (MSDF 2008/56/EC); with Descriptor 10 aiming for: “Properties and quantities of marine litter do not cause harm to the coastal and marine environment” (MSFD 2008/56/EC). On that basis and in addition to the “Regional Action Plan for Marine Litter in the Baltic Sea” (HELCOM 2015), there are legal obligations to record and reduce the marine litter pollution of the various marine habitats in the Baltic Sea (LUNG-MV 2015). A joined and harmonized monitoring strategy (JRC, 2011) was adapted from the OSPAR Guideline (OSPAR, 2010) and further developed, ensuring that data is comparable among monitoring surveys. This bare-eye method primarily focuses on stretches of sand or gravel beaches at least 1 km long, with surveys of 100 meters, and targets macro-litter (>25 mm). Long-term surveys such as the MARLIN Project “Baltic Marine Litter” (MARLIN 2011) of the marine litter of Estonian, Latvian, Swedish, and Finnish coasts have been conducted to meet these requirements. Beach surveys in Germany and Lithuania following OSPAR Guideline (OSPAR 2010) took place (Schernewski et al. 2018; Haseler et al. 2019, 2020). To gather more knowledge about meso- (5–25 mm) and large micro-litter (2–5 mm), different sieving (i.e., Rake method) and bare-eye methods were used at Baltic beaches (Haseler et al. 2018, 2019). However, these approaches focused on the open coastal beaches only, not considering the shores of the inner-coastal waters such as lagoons and estuaries.

The total Baltic Sea catchment area is four times larger than the surface area of the Baltic Sea, and it comprises nearly 1.8 million km^2^ (Räike et al. 2021). More than 85 million people live in the Baltic Sea catchment area. The catchment area of those rivers is covered by agricultural fields, which substantially increases the pollution load (Schernewski et al. 2021). Supporting Source-to-Sea Framework for Marine Litter Prevention by Granit et al. (2017), we believe that lagoons and estuaries as transitional zone could be an essential provider of information about river basin-related litter leakage. During the ice melting, its motion in the spring (Idzelytė et al. 2019), and accumulation on the coastline of coastal lagoons and estuaries, bigger litter pieces might be fragmented into smaller ones. However, it is unclear whether lagoons and coastal estuaries play a role as a sink, transition zone, or micro-litter source. Furthermore, the knowledge of pollution of lagoons is scarce worldwide.

To investigate plastic litter at coastal marine beaches, methods such as OSPAR 100 m transect are most often used (Schulz et al. 2015; Simeonova et al. 2017; Schernewski et al. 2018; Falk-Andersson et al. 2019; Haseler et al. 2020). However, the widely used OSPAR method for pollution of lagoons and estuaries assessment cannot be applied because beaches of lagoons and estuaries are mostly not long enough to survey a 100 m transect. Furthermore, this method does have a weakness in tackling meso- (5 – 25 mm) and large micro-litter (2 – 5 mm) (European Commission 2013). So far, few studies on coastal lagoon pollution (Oztekin et al. 2020; Velez et al. 2020) have been conducted, and most of them used methods applicable to long, sandy beaches. Therefore, a new method for all sizes of litter investigation in the inner-coastal waters needs to be established. This method needs to meet specific requirements, such as (i) it needs to be low-cost and straightforward to enable monitoring of many locations; (ii) it should be cost-efficient - meet the demand of authorities to keep costs low; (iii) it should be suitable to involve trained laymen (citizen science) and with a possible benefit to serve for environmental awareness rising; (iv) litter monitoring method and the gained data should be suitable to be combined or compared with the data of the OSPAR 100 m beach monitoring method (OSPAR 2010) and/or “Joint List of Litter Categories for Marine Macrolitter Monitoring” (Fleet et al. 2021) to enable comprehensive pictures of the state of pollution of coastal and marine waters; (v) it should allow an analysis of litter sources to support and enable mitigation measures; (vi) it needs to be generally applicable around the Baltic and in Europe to meet the requirements of Descriptor 10 of the MSFD.

The objectives of this study are: (a) to develop a methodology suitable for large micro-, meso-, and macro-litter monitoring at sandy coastal strips of lagoons and estuaries; (b) to test this method in a wide range of Baltic lagoons and estuaries to get an overview of present pollution state; (c) to analyze the composition, type, and abundance of beach litter (d) to assess significant pollution sources and address the extent to which lagoons serve as a sink for river-borne and land-based litter; (e) to discuss this methodology’s applicability and suitability concerning expanding and complementing existing beach litter monitoring methods towards transitional waters and inner-coastal waters.

## Material and Methods

### Study Area

**Fig. 1:**
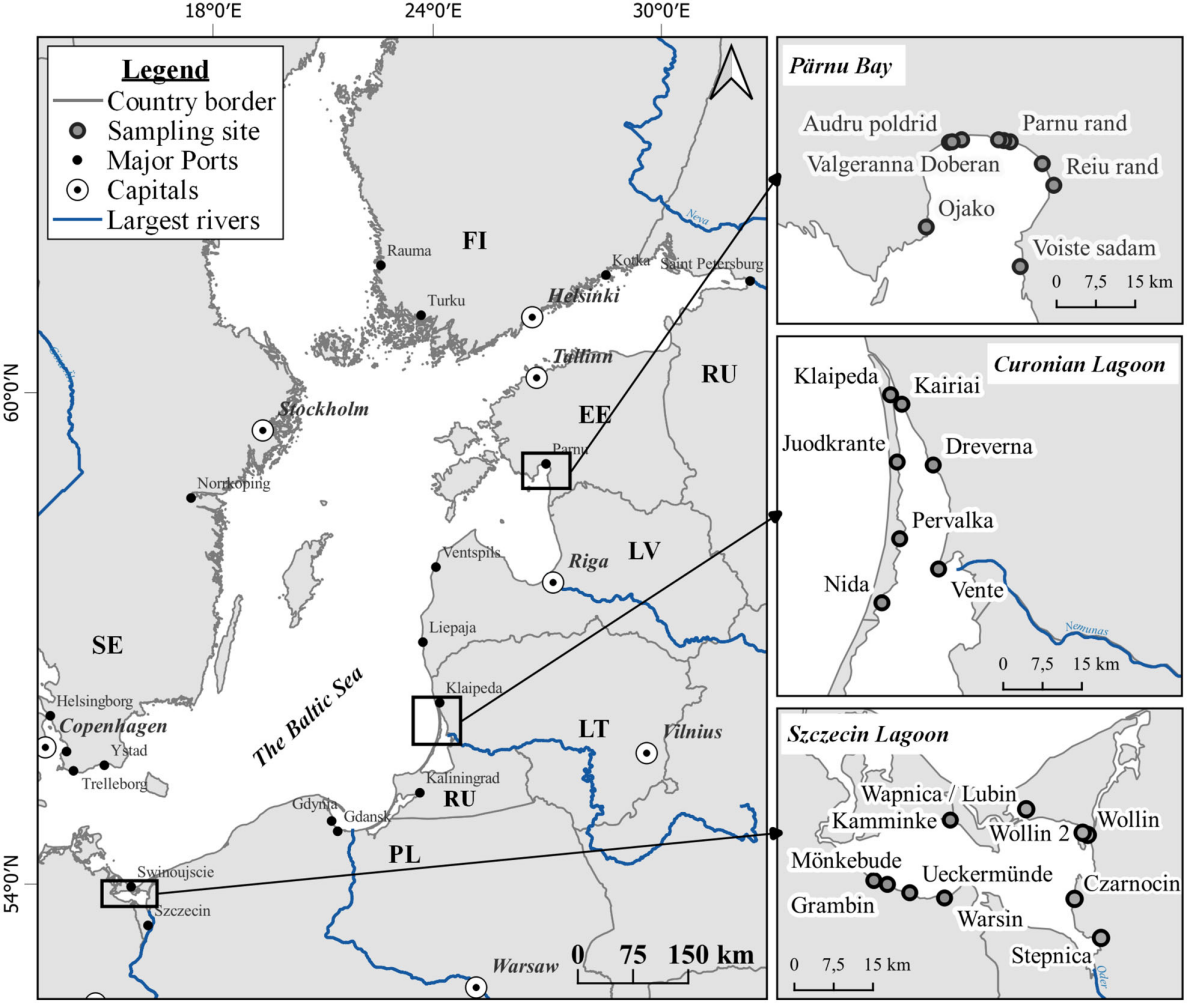
Map of the study areas in 2018: Pärnu Bay (EE- Estonia), Curonian Lagoon (LT-Lithuania), and Szczecin Lagoon (DE – Germany)

To investigate litter pollution (>2 mm) on beaches of inner-coastal waters, two lagoons and one bay with at least six sampling sites were chosen for this study. Sampling campaigns were conducted in the summer of 2018 (Fig. 1). The Nemunas and the Oder river are the two major rivers of the southern Baltic Sea catchment area. They are connected to the two largest lagoons (the Curonian and Oder lagoons), contributing to the most significant share of the nutrients to the Baltic Sea. Therefore, it is the primary source of pollution (Čerkasova et al. 2018). Lagoon-type bays such as Pärnu represent this study’s highly recreational and urbanized areas.

*The Curonian Lagoon* is the largest transboundary coastal lagoon connected to the south-eastern Baltic Sea via the narrow strait of Klaipeda (Lithuania). The lagoon is divided by the state’s borders, the Kaliningrad region (Russia), and Lithuania. The southern part is a freshwater body where the wind determines the hydrological regime. Meanwhile, the north part of the lagoon is mainly influenced by the Nemunas river discharge and inflows of the brackish Baltic sea waters (Gasiūnaitė et al. 2008). The Nemunas River provides more than 90% water and nutrient input into the lagoon. It also plays a role as a transition area for sediment transport.

*The Szczecin Lagoon—Oder/Odra Lagoon* is located in the southern Baltic Sea and is divided by the border between Germany and Poland. The Szczecin Lagoon is connected to the Baltic Sea via three outlets. The River Odra is the main freshwater inflow to the lagoon, contributing at least 94% of the lagoon’s water budget (Radziejewska and Schernewski 2008). The Szczecin Lagoon is also a shallow water body, with varying salinity from 0 at the central part to 6 PSU at the Swina Channel, the main channel for saline water intrusions.

*The Pärnu Bay* is a shallow, semi-enclosed water basin and a vital recreation area located at the northeastern coastline of the Baltic Sea facing the Gulf of Riga (Kotta et al. 2008). The hydrological conditions of the bay are influenced mainly by meteorological processes, the discharge of the Pärnu River, and the exchange of water masses with the open part of the Gulf of Riga. The Pärnu River catchment area is highly agricultural and is responsible for 10% of riverine inflow into the Gulf of Riga. Together with Pärnu town, these are the primary sources of pollution (Kotta et al. 2004). It is known that about 85% of the waste and stormwater of Pärnu town is discharged via a deep-sea outlet into Pärnu Bay (Reihan 2021).

### Developed Sampling Scheme for Beaches of Inner-Coastal Waters

#### Macro-litter (>25 mm)

**Fig. 2:**
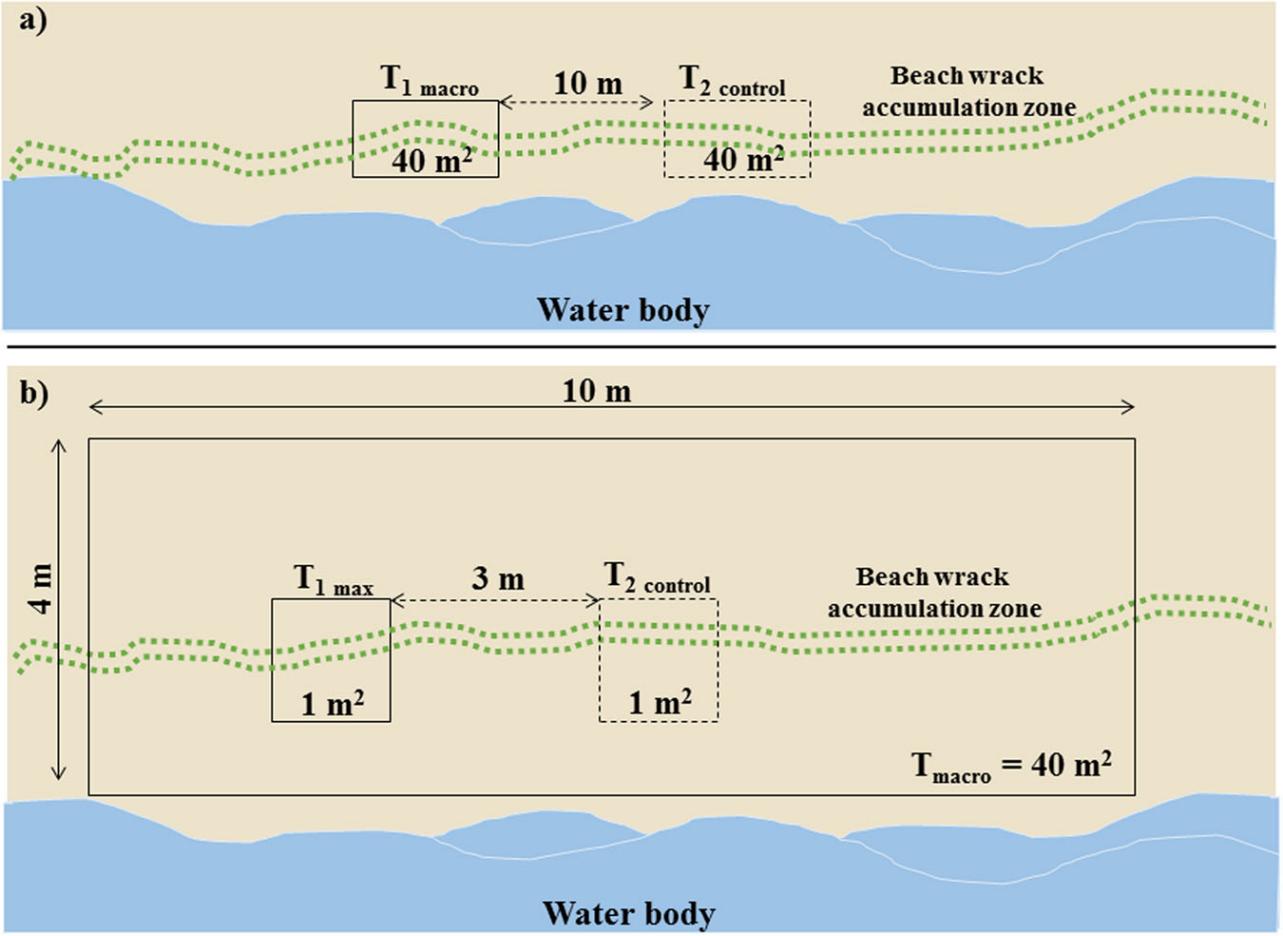
Developed sampling scheme: **a** visualization of a two 40 m^2^ rectangular polygons (T_1 macro_ and T_2 control_) located at the beach wrack accumulation zone; **b** visualization of a two 1 m^2^ squares (T_1 max_ and T_2 control_) within 40 m^2^ rectangular polygons

A visual survey of beached macro-litter was performed in a 40 m^2^ (4 × 10 m) rectangular polygon (Fig. 2a) along the waterline, including the tidal accumulation zone. The area was systematically walked up and down (parallel to the waterline), and all litter pieces visible to the naked eye were collected and counted, according to the “Joint List of Litter Categories for Marine Macrolitter Monitoring” (Fleet et al. 2021). Control sampling was done at a distance of 10 m along the waterline from any already sampled transects. At each sampling site, at least one additional control transect was monitored to prove we were not influencing the results by choosing the transect location.

#### Meso- and large micro-litter (2–25 mm)

Two 1 m^2^ squares within the macro-litter survey site were sampled for meso- (5–25 mm) and micro-litter (2–5 mm). The first square T_1 max_ (Fig. 2b) was placed in what appeared to be the most polluted area along the beach accumulation zone. The second square T_2 control_ was set 3 meters away (from the edge of the T_1 max_ square). For the sampling of meso- and large micro-litter, a 2 mm mesh-size metal sieve, a metal spatula, and a bucket were used. The top 2 cm of beach/coast sediment was collected with a metal spatula and placed in a metal sieve. Sieve was carefully (not to be wholly submerged) placed in a bucket half full of water and manually shacked until all the sand was sieved out. The procedure was repeated until all the surface sand from the sampling square was sieved. All litter pieces were retained from the sieve tray and placed in labeled plastic bags.

Later, all size litter pieces were photographed, counted, measured, and classified according to the Master list (Haseler et al. 2020) with the adaptations of categories of litter items initially based on (European Commission 2013).

### Matrix Scoring Technique for Beach Litter Sources

The adapted Matrix Scoring Technique determined potential sources of litter items (Tudor and Williams 2004). This method is based on likelihoods, which consider the possibility that specific litter items may originate from more than one source. At first, all litter pieces from all study areas found in the 40 m^2^ survey area, including squares (2 × 1 m^2^), were sorted to the top litter items most often obtained. Next, for every single litter item, a score related to the possible source was given. The different likelihood scores are: not considered/impossible (0), very unlikely (1), unlikely (2), possible (3), likely (4), and very likely (5). This method also uses a percentage allocation, assigning each litter item to several possible sources (Table 1). In this study, we used nine potential sources of litter that have been previously described by The Regional Planning and Environmental Research Group of Germany (ARSU 2019): (i) fishing and fishing ports; (ii) leisure-, pleasure, recreational boating and ports; (iii) passenger- and cargo shipping; (iv) offshore industry; (v) passenger- and cargo ports; (vi) tourism and recreational activities; (vii) littering and waste management; (viii) wastewater treatment and stormwater drainage; (iv) coastal infrastructures, industry, and trade (Supplementary 1).

**Table 1:** The probability phraseology describes the likelihood of a litter item originating from a possible source. The likelihood, i.e., 90%, is based on expert judgment

Probability phraseology	Score	%	Score description
Very likely	5	90	It is very likely that litter items will be entered in very large quantities
Likely	4	50	It is likely that litter items will be entered in large quantities
Possible	3	15	It is possible that litter items will be entered in relevant quantities
Unlikely	2	5	It is unlikely that litter items will be entered in relevant quantities, it cannot be excluded, however
Very unlikely	1	1	It is very unlikely that litter items will be entered in relevant quantities, it cannot be excluded, however
Not considered/impossible	0	0	It is excluded/impossible that litter items will be entered

The source allocation of the most common litter items was done by experts working in the marine litter pollution field from three countries: Germany, Estonia, and Lithuania.

### Costs of the Lagoon Litter Monitoring

Costs of the established method for litter monitoring of the inner-coastal waters were calculated considering the:

(i) Implementation costs that include investment in fixed assets such as Microphazir PC (by Analyticon, Germany) needed for analyzes of polymer type, as well as a 2 mm mesh size sieve to obtain micro- and meso-litter from 1 m^2^ squares (Fig. 2), other tools such as trowels, measuring tape, buckets, and training workshop for the staff;
(ii) Annual running costs include fieldwork and laboratory work, including measuring, sorting out litter items, polymer analyses, data processing, and reporting. Furthermore, this calculation of the costs follows the MSFD guidelines and shall compare with other methods (European Commission 2013). This study recommends monitoring one beach per 100 km^2^ of a water body. For example, annual running costs for fieldwork were calculated for monitoring seven beaches of Szczecin Lagoon (~687 km^2^) four times a year. The hours indicated for the travel to the beach and back, field, and laboratory work is based on our own experiences. The annual staff hours and costs were calculated for seven beaches monitored four times per year using the staff salary pay scale of the federal state authority of Germany (37.5 € per hour according to the German pay scale group E9 Level 1) (Haseler et al. 2020).

### Statistical Analyses

Descriptive and statistical analyses were performed using the R Studio (Rcmdr commander) and XLSTAT software (XLSTAT 2021). Before the analysis, the normality of variables was tested using the Kolmogorov–Smirnov test. The data deviated from the normal probability distribution. Therefore, the non-parametric Kruskal–Wallis test was applied to compare differences in the litter densities obtained in 40 m^2^ polygons and heavily polluted and controlled 1 m^2^ squares. In contrast, the Kruskal–Wallis test for pair-wise comparison of *k* samples was applied to compare litter densities among inner-coastal water bodies. Results were considered significant at a *p*-value less than 0.05.

## Results

### General Results of Litter Distribution in Inner-Coastal Waters of the Baltic Sea

In total, 23 beaches from 3 inner-coastal waters of the Baltic Sea were investigated for all sizes of litter pollution in 2018. In two major Baltic Sea lagoons and one bay, 817 litter pieces (57 different litter items) were found. Micro- and meso-litter size categories resulted in much higher numbers per m^2^ than macro-litter pieces across all lagoons and bays. The total number of litter pieces per water body varied from 122 in Pärnu Bay to 563 pieces in the Curonian Lagoon, thus, indicating substantial differences in the total number and densities of litter between the water bodies. The average density of litter pieces found in 40 m^2^ polygons was 0.22 pieces/m^2^ ± 0.55, with a median of 0.10 (Table 2). The amount of litter found significantly differed among the inner-coastal waters (*p* < 0.05; *n* = 55) but not between polygon T_1 macro_ and polygon T_2 control_ samplings (*p* > 0.05; *n* = 55). Altogether, a heavily polluted 1 m^2^ square resulted in an average litter density of 3.66 pieces/m^2^ ± 4.84, a median of 2.0 pieces/m^2^. In contrast, control squares had a similar average density of 2.01 pieces/m^2^ ± 2.58, median 1.00 (Table 2), and did not differ significantly from heavily polluted ones (*p* > 0.05; *n* = 119). However, litter density obtained from 1 m^2^ square significantly differed among inner-coastal waters (*p* > 0.05; *n* = 119).

**Table 2:** The average beach litter density for the studied inner-coastal waters in mean numbers of litter pieces/ m^2^ ± SD and median in 40 m^2^ rectangular polygons, and T_1 max_ and T_2 control_ squares within polygons

Country	Lagoon/Bay	Mean number of litter pieces/m^2^ ± SD and median per area	Mean number of litter pieces/m^2^ ± SD and median in T_1_ max	Mean number of litter pieces/m^2^ ± SD and median in T_2_ control
Germany/Poland	Szczecin Lagoon	0.12 ± 0.08; 0.10	1.25 ± 1.48; 1.00	0.70 ± 1.23; 0.00
Lithuania	Curonian Lagoon	0.53 ± 0.99; 0.23	6.95 ± 6.22; 5.00	4.26 ± 3.55; 3.00
Estonia	Pärnu Bay	0.06 ± 0.08; 0.03	2.60 ± 3.88; 1.00	1.05 ± 1.24; 1.00
Total all lagoons and bays		0.22 ± 0.55; 0.10	3.66 ± 4.84; 2.00	2.01 ± 2.58; 1.00

Almost half of the total pieces (370 pieces, 45.29%) found in all studied locations around the Baltic Sea were artificial polymer material, 208 pieces (25.46%) of cigarette butts, and 159 (19.46%) glass and ceramics. In contrast, 9.79% of litter pieces were attributed to metal, paper/cardboard, paraffin, and other litter categories. The top 10 litter items of all inner-coastal waters contributed 77.4% (632 pieces) of the total litter amount. The most common litter items from all water bodies were cigarette butts (208 pieces, 25.4%), non-identifiable meso-litter pieces (126 pieces, 15.4%), other glass items (84 pieces, 10.2%), and micro-litter pieces (53 pieces, 4.3%). The rest, 19.7% (161 pieces), were bottles, including pieces, macro-litter pieces, construction material (bricks, cement, pipes), industrial pellets, and small plastic bags, including pieces and paper fragments (Table 3).

**Table 3:** Top ten litter items per inner-coastal waters and total cumulative for all studied areas

	All lagoons and bays (total)	Szczecin Lagoon (*n* = 6)	Curonian Lagoon (*n* = 7)	Pärnu Bay (*n* = 10)
	Total litter pieces = 817	Total litter pieces = 132	Total litter pieces = 563	Total litter pieces = 122
1	Cigarette butts and filters	Construction material (bricks, cement, pipes)	Cigarette butts and filters	Other glass items
	208/25.4%	21/15.9%	188/33.4%	23/18.9%
2	Plastic pieces >2–25 mm (meso)	Cigarette butts and filters	Plastic pieces > 5–25 mm (meso)	Plastic pieces > 5–25 mm (meso)
	126/15.4%/40.9 %	18/13.6%/29.5%	108/19.2%/52.6%	14/11.5%/30.3%
3	Other glass items	Other glass items	Plastic pieces 2–5 mm (micro)	Bottles incl. pieces
	84/10.2%/51.2%	18/13.6%/43.2%	48/8.5%/61.1%	13/10.7%/41.0%
4	Plastic pieces 2–5 mm (lg. micro)	Bottles incl. pieces	Other glass items	2721
	53/6.5%/57.6%	11/8.3%/51.5%	43/7.6%/68.7%	9/7.4%/48.4%
5	Bottles incl. pieces	Plastic caps/lids drinks	Industrial pellets	Plastic pieces > 25 mm (macro)
	35/4.3%/61.9%	5/3.8%/55.3%	26/4.6%/73.4%	7/5.7%/54.1%
6	Plastic pieces >25 mm (macro)	Plastic pieces >2–25 mm (meso)	Plastic pieces >25 mm (macro)	String and cord (diameter < 1 cm)
	35/4.3%/66.2%	4/3.0%/58.3%	24/4.3%/77.6%	6/4.9%/59.0%
7	Construction material (bricks, cement, pipes)	Plastic pieces >25 mm (macro)	Tangled nets/cords	Paraffin/Wax micro
	31/3.8%/70.0%	4/3.0%/61.4%	13/2.3%/79.9%	6/4.9%/63.9%
8	Industrial pellets	Foil wrappers, aluminum foil	Paper fragments	Oil/Tar/Paint particles
	26/3.2%/73.2%	4/3.0%/64.4%	12/2.1%/82.1%	6/4.9%/68.9%
9	Small plastic bags, incl. pieces	Small plastic bags, incl. pieces	Bottles incl. pieces	Plastic pieces 2–5 mm (micro)
	18/2.2%/75.4%	3/2.3%/66.7%	11/2.0%/84.0%	5/4.1%/73.0%
10	Paper fragments	Other metal pieces < 50 cm	Small plastic bags, incl. pieces	Small plastic bags, incl. pieces
	16/1.9%/77.4%	3/2.3%/68.9%	11/2.0%/86.0%	4/3.3%/76.2%
	632/77.4%	91/68.9%	484/86%	93/76.2%

### Study Area-Specific Litter Abundance

#### Curonian lagoon

**Fig. 3:**
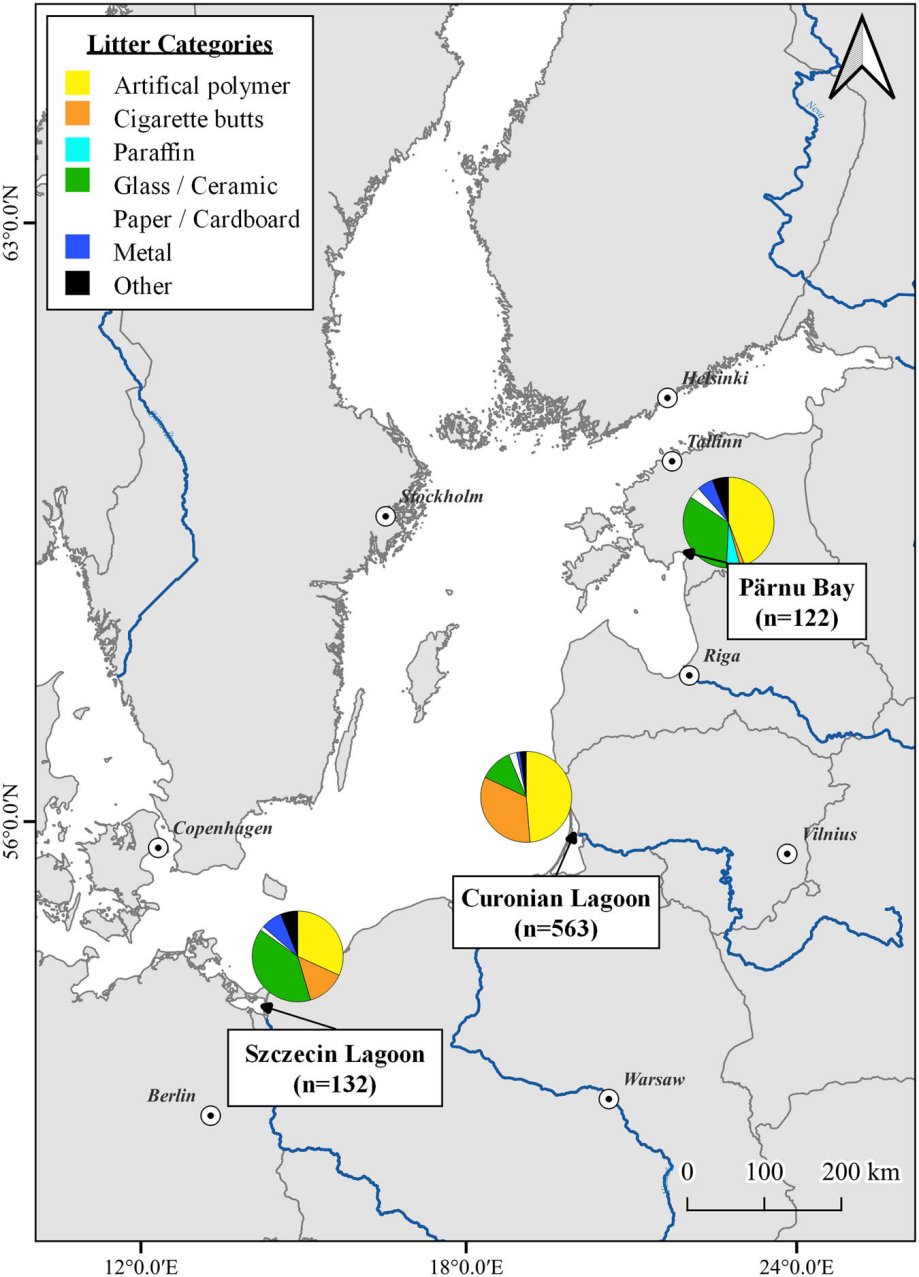
Litter categories in total numbers (micro-, meso-, and macro-; *n* – total number of litter pieces) per inner-coastal waters of the Baltic Sea: Pärnu Bay, Curonian Lagoon, and Szczecin Lagoon

Seven beaches along the Lithuanian coast of the Curonian Lagoon were investigated for micro-, meso- and macro-litter distribution. Altogether, 563 litter pieces were found, which were distributed as follows: 227 (40.3%) micro- and meso-litter pieces and 336 (59.7%) macro-litter pieces. The number of litter pieces found in 40 m^2^ polygons in the Curonian Lagoon varied from 0.05 to 4.07 pieces/m^2^; on average, it was 0.53 ± 0.99 pieces/m^2^ (Table 2). The mean number of litter pieces in the T_1max_ m^2^ square was 6.95 ± 6.22 pieces/m^2^, while in a control square, 4.26 ± 3.55 pieces/m^2^ were found. The density of all-sizes litter found on the beaches of the Lithuanian side of the Curonian lagoon was 0.93 pieces/m^2^. Of all litter pieces found, 48.66% were assigned to the artificial polymer litter category. Followed by cigarette butts, glass/ceramics, and paper/cardboard, with 33.39%, 11.72%, and 2.84%, respectively (Fig. 3). The top 10 litter pieces found on the Curonian Lagoon beaches summed up to 484 pieces representing 86% of the total litter amount, and are listed in Table 3.

#### Szczecin lagoon

In the Szczecin Lagoon, ten beaches (five in Germany and five in Poland) were sampled for all sizes of litter pollution. Out of 132 pieces found, 29.5% (*n* = 39) of pieces were attributed to micro- and meso-litter category while 70.5% (*n* = 93) to macro-litter. The number of litter pieces found in 40 m^2^ rectangular polygons varied from 0 to 0.28 pieces/m^2^ with an average of 0.12 ± 0.08 pieces/m^2^. The mean number of litter pieces in a heavily polluted 1 m^2^ square was 1.25 ± 1.48 pieces/m^2^, almost two times more than in a control square (mean 0.70 ± 1.23 pieces/m^2^) (Table 2). The glass/ceramics litter category was the most common of all litter pieces found; it contributed 52 pieces (39.39%), while artificial polymer materials and cigarette butts resulted in 42 (31.82%) and 18 (13.64%) pieces, respectively. The rest, 15.15%, were metal, paper/cardboard, and other litter (Fig. 3). The top 10 litter items found in the Szczecin Lagoon represented 68.9% of total litter pieces (Table 3).

#### Pärnu bay

A total of 122 litter pieces were found at ten coastal beaches of Pärnu Bay, and they were distributed to 73 (59.8%) micro- and meso-litter and 49 (40.2%) macro-litter pieces. Litter densities observed in 40 m^2^ polygons varied from 0 to 0.28 pieces/m^2^ with an average of 0.06 ± 0.08 pieces/m^2^ (Table 2). The mean number of litter pieces in heavily polluted 1 m^2^ squares (T_1 max_) was 2.60 ± 3.88 pieces/m^2^, while in a control square, 1.05 ± 1.24 pieces/m^2^. Artificial polymer (54 pieces) and glass/ceramic (41 pieces) items presented 77.87 % of the total items found (Fig. 3). Top 10 litter pieces found on the Pärnu Bay beaches summed up to 93 pieces (76.2%) of the total number (Table 3).

### Analysis of all Size Litter Sources

**Fig. 4:**
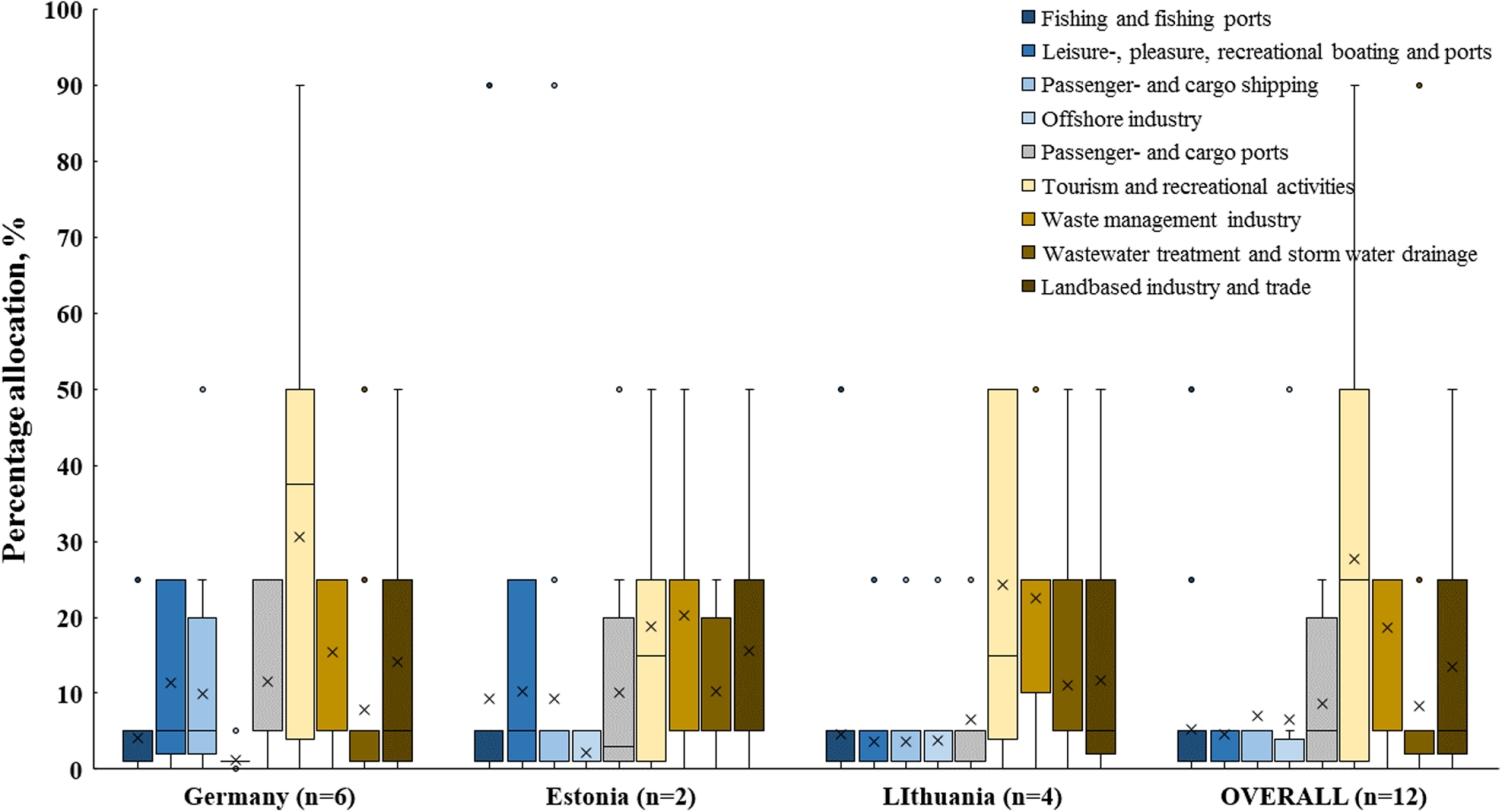
The Box and Whisker plot shows the percentage allocation of experts from different countries and overall results for the top 25 litter items; *n* – the number of experts that participated in source allocation; dots indicate outliers; *x* – the mean marker; the colored box is defined by lower and upper quantile including median; whiskers shown in the grey line indicate variability outside the upper and lower quartiles

In total, 12 experts (six from Germany, two from Estonia, and four from Lithuania) participated in the scoring (The Matrix Scoring Technique by Tudor and Williams (2004)) of the most common litter items found at the coast of all studied water bodies. The differences in perception of source allocation are shown in Fig. 4. The experts from different countries have a similar perception of four possible litter sources’ “Waste management industry”, “Land based industry and trade”, “Fishing and fishing ports”, and “Offshore industry”. “Tourism and recreational activities” received the highest scores and, therefore, percentage allocation in German and Lithuanian expert groups, except for the Estonian one. Lithuanian group of experts gave lower scores than Estonian and German groups for two possible litter sources “Leisure-, pleasure, recreational boating, and ports”, and “Passenger- and cargo shipping”. While a German group of experts gave a lower score for “Wastewater treatment and storm water drainage”. The average sum of all possible sources for all litter items varied among the expert groups from 96% (LT group) to 106% (DE group). The entire perception of all 12 experts ended up with a 99% chance that all litter items come from 9 sources (Fig. 4).

**Fig. 5:**
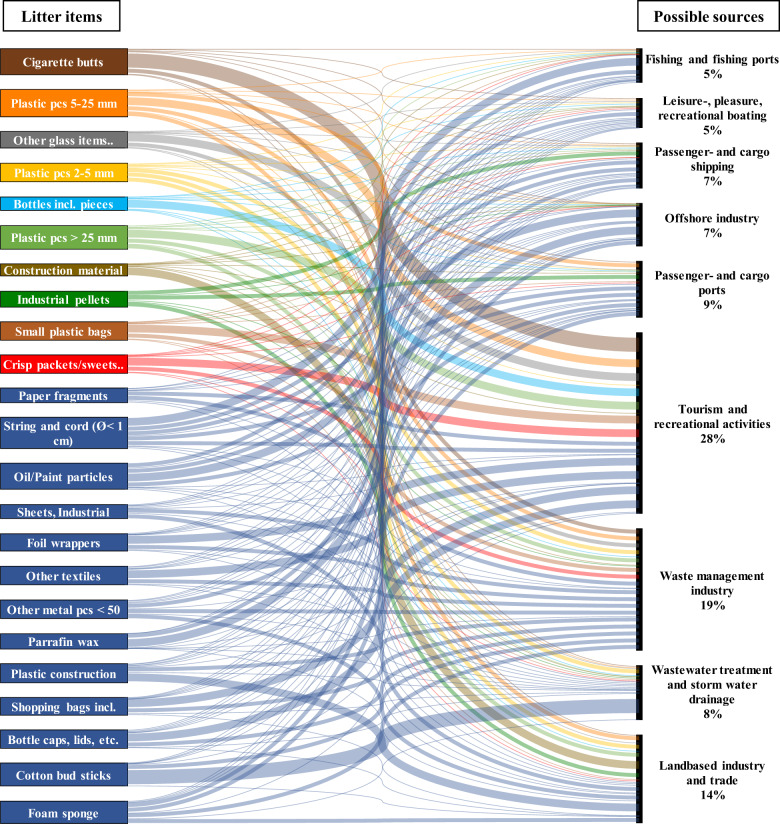
The Sankey diagram summarizes the possible sources of litter items based on source allocation done by 12 experts from different countries around the Baltic Sea

The Sankey diagram (Fig. 5) shows the overall litter allocation to their potential sources in detail. Here, we see that majority of the most common litter items such as “Cigarette buts”, “Crisp packets/sweets”, “Small plastic bags”, “Bottles including pieces”, and others were attributed to “Tourism and recreational activities” with a higher likelihood. Litter items such as “Strings and cord, Ø < 1 m”, and “Cotton bud sticks” were attributed to the major and most likely sources of “Fishing and fishing ports” and “Wastewater treatment and stormwater drainage”, respectively. While “Construction material” and “Plastic construction waste” were attributed to the “Land based industry and trade” source. Unidentified litter pieces of micro-, meso-, and macro-size were attributed with a higher likelihood to come from “Tourism and recreational activities” and an equal possibility to come from all other possible sources.

Further analysis of source allocation was done with an overall score retrieved from all 12 experts and the amount of litter found. The source allocation results indicate that the highest share of most common litter items found in all studied sites, with a contribution of 19.6%, came from “Tourism and recreational activities”. As many as 15.1% and 11.6% of most commonly found litter items are likely to come from the “Waste management industry” and “Land based industry and trade”, respectively. About 11% of litter contributes to “Passenger and cargo ports”. The smallest share of the most common litter found was attributed to the “Offshore industry” source (Supplementary 2).

### Cost Estimate

The estimation of time and costs was done based on the developers’ experience of the method obtained during the surveys of this study and federal state authority staff salaries (37.5 € per hour). The cost per survey (one beach) done by the expert would cost around 400 €. Meanwhile, monitoring seven beaches (1 beach for 100 km^2^ of a water body) four times a year, including field and laboratory analysis, would cost approximately 12,000 € a year. Altogether, implementing a new method for monitoring litter pollution in the inner-coastal waters (the first investment and annual cost for the salary of one expert) costs 35,000 € (Table 4).

**Table 4:** Estimation of costs of the inner-coastal water monitoring method developed in this study includes investigation of all litter sizes.

Work step	Inner-coastal waters			
	Hours [h] for one survey	Costs [€] for one survey	Annual hours^d^ [h year -1]	Annual cost^d^ [€ year-1]
Investment in the implementation^a^	21,500			
Annual field costs^b^		206		5775
Travel by car to the beach and back	3	112.5	84	3150
Bare-eye survey (2x~40 m^2^) at one beach	1	37.5	28	1050
Sieving (4 x 1 m^2^) at one beach	1.5	56.3	42	1575
Annual lab costs^c^		187.5		5250
Litter analysis steps	2.5	93.8	70	2625
Data processing	1.5	56.3	42	1575
Reporting	1	37.5	28	1050
Running material expenses		20		100
Total sum		~400		~12,000

Annual hours of one person and costs calculated for monitoring seven beaches four times a year. Federal state authority staff salaries (37.5 € per hour)

^a^includes investment in fixed assets such as Microphazir (21,000 €, lifetime five years) and sand sieve; training workshop for staff

^b^includes travel to the site and sampling according to the methodology described in Material & Methods

^c^includes litter analysis (counting, measuring, categorizing, polymer analysis), data processing, and reporting

^d^annual hours and costs were calculated for seven beaches monitored four times per year

## Discussion

This study investigates the two largest lagoons in the Baltic Sea and one of the largest bays located in the northeastern part of the sea for all-sizes litter pollution. The entire Szczecin Lagoon, the Lithuanian side of Curonian Lagoon, and parts of Pärnu Bay have been declared as Birds Directive Sites, Habitats Directive sites, or both Birds and Habitats directive sites. Thus, studied lagoons and bays are essential to ensure the long-term survival of Europe’s most valuable and threatened species and habitats (Kotta et al. 2008; Radziejewska and Schernewski 2008; Povilanskas et al. 2014). Furthermore, they have tremendous economic value. Tourism, fisheries, and port activities are the main socio-economic activities in the considered areas (UNEP 2016; Inácio et al. 2018). Lagoons of the Baltic Sea face similar problems, with eutrophication being the main (Nehring 1992; Raateoja and Setälä 2016); however, knowledge about litter pollution in such water bodies is scarce. Many large Baltic rivers do not enter the sea directly but pass lagoons and estuaries (bays). Therefore, lagoons and bays can be regarded as pollution accumulation zones.

This study’s average macro litter density varied from 0.06 pieces/m^2^ in the Pärnu Bay to 0.53 pieces/m^2^ in the Curonian Lagoon, comparable to the other lagoons in Europe: mean density in Sarıkum Lagoon (the Black Sea) was 1.51 ± 0. 57 pieces/m^2^ (Oztekin et al. 2020); Ria Formosa Lagoon (the Atlantic Ocean) from 0.12 ± 0.01 pieces/m^2^ to 9.10 ± 2.05 pieces/m^2^ (Velez et al. 2020).

Comparing litter densities per 1 m^2^ found on the coastal beaches and lagoon sites, we see that, i.e., in Lithuania, the density of litter in 1 m^2^ is the same on the lagoon (0.93 pieces/m^2^) as on the coastal beaches of the Baltic Sea (0.93 pieces/m^2^) (Haseler et al. 2020). However, litter densities in Szczecin Lagoon were lower than those reported for the coast of the German Baltic Sea (Haseler et al. 2018). There are several possible explanations for these results. Firstly, lagoon beaches are less visited than coastal ones (Haseler et al. 2018; Kataržytė et al. 2019); therefore, they are less polluted. Secondly, the fate of litter items in the lagoon depends on water exchange between the lagoon and the sea: if the river outflow is more intensive, litter is washed out on the coastal beaches.

One of the investigated transboundary lagoons in this study – the Szczecin Lagoon, indicates that the amount of litter found on the beaches on Germany’s side of the lagoon was precisely the same as on the Poland side of the lagoon – 0.16 pieces/m^2^. Litter circulates and distributes within the lagoon if outflow from the river is less intensive.

Investigating the top 10 litter items found in this study’s lagoons and bays, we recognized several items to be very different from the top 10 litter from the coastal beaches around the Baltic Sea (Haseler et al. 2020). The main difference in the top litter items was the paraffin (micro- and meso-size) which is washed out of the ships in the harbors and often ends up in the open sea (outside the 12-mile zone). Therefore, it is found in high densities on the coastal beaches, including the beaches of Pärnu Bay investigated in this study. In the meantime, paraffin was not present among the top 10 litter items on the beaches of two lagoons investigated in this study. On the other hand, two out of the top 10 litter items found in this study, “Construction material” and “Plastic construction material” were allocated by the experts to the “Land based industry and trade”. It means that lagoons and bays could be potential sink of land-based litter through the connection of the major rivers. River runoff as the primary source of litter (22%) was also indicated in the study performed on the SE Black Sea beaches (Aytan et al. 2020). Sewer overflows and stormwater were of the highest importance for large micro- and meso-litter emissions in the south-eastern Baltic Sea estuary of Warnow (Schernewski et al. 2021). Further supporting our findings, the study case of Ria Formosa (Portugal) indicated that construction material (heavier materials like ceramics, glass, and metal) derived from land-based sources was dominant in the lagoon compared to the open sea coastal sites (Velez et al. 2020).

### Strengths and Weaknesses of Methodology for Litter Monitoring of Coastal Lagoons

Our results showed that the monitoring method of litter at the coastal lagoons and bays could determine micro, meso, and macro-litter. An evident strength of the methodology established in this study is relatively low-costs and time efficiency. Initial costs to implement this methodology are lower than a UAV methodology (Escobar-Sánchez et al. 2021), somewhat equal to the Sand-rake method costs (Haseler et al. 2020), and slightly higher than OSPAR. The annual running costs of the lagoon litter monitoring method are also much lower than those of the previously mentioned methods (OSPAR, UAV, and Sand-rake) due to the fewer hours needed for field activities. Surveying two polygons of 50 m^2^ by the Sand-Rake way takes twice as much time (5 h) (Haseler et al. 2020), whereas investigating two polygons of 40 m^2^ by the methodology established in this study—takes 2.5 h. In contrast to the Sand-Rake method, it is applicable when the sediment is wet.

In general, the lagoon litter monitoring methodology was established to be implementable for volunteer-based monitoring, which would significantly decrease annual running costs. This method is easy to explain and does not require fancy tools to apply. It would also promote community-engaged citizen science, at least in the field activities, and allow litter pollution investigation at a smaller scale of lagoons and bays. However, the perception of litter pollution differs among people and depends on various socio-demographic factors (i.e., age, income level, educational background, and gender) (Rayon-Viña et al. 2018). The difference in perception, especially for hardly visible or small objects, could be an additional challenge for litter data integrity. For all that, we recommend that every litter monitoring activity is complemented with the polymer-type analysis done using MicroPhazir hand-held device (or similar instrument/equipment). While items are listed according to the “OSPAR Marine Litter Monitoring Survey Form”. Furthermore, before implementing volunteer-based monitoring of litter pollution at coastal lagoons and bay beaches, training on methodology given by an expert should take place.

The main idea to establish a new method for coastal lagoon litter pollution was driven by the fact that the OSPAR 100 m monitoring methodology could not be applied due to relatively short lagoon beaches and the loss of litter below 25 mm in size. Moreover, the Sand-Rake method proposed by Haseler et al. (2018) could not be used due to the granulometry of lagoon beaches and wrack accumulation, which would complicate the sieving of sediments for smaller-size litter. However, the data obtained by a new method must be suitable to combine with the data of a 100 m method by OSPAR. For example, the average density of all-sizes litter pieces in Pärnu Bay was determined as 0.28 pieces/m^2^ using the Sand-Rake method (Haseler et al. 2020) and 0.06 pieces/m^2^ using the methodology established in this study. Furthermore, nine out of ten top litter items found along the Estonian coast of the Baltic Sea (Haseler et al. 2020) were also obtained using the newly established methodology of this study, meaning that this study’s results are comparable with the results of the Sand-Rake method. In the meantime, the results of large micro- and meso-litter are also reliable, as the sieving is comparable to the Frame method (9 m^2^) and should have similar recovery results (Haseler et al. 2018). Furthermore, we assume that the macro-litter results of 40 m^2^ are more reliable than the 100 m OSPAR results as a much smaller area is investigated. Regarding monitoring sites, we recommend surveying one sampling site for 100 km^2^ of a lagoon or bay area. Choosing sampling sites, the length of a beach (enough to sample two replicates of 40 m^2^) should be preferred. Beaches that are recognized as official bathing sites and sites that are possibly affected by urbanization or port activities should be considered, too.

Although no statistically significant amounts of litter between seasons were found in several studies (Balčiūnas and Blažauskas 2014; Schernewski et al. 2018; Oztekin et al. 2020), the OSPAR Guidelines suggest evaluating the trend of litter abundance every three months. Based on this suggestion, calculations of litter monitoring costs were done considering the same frequency of litter monitoring (four times a year). In addition, event-based (i.e., storms, heavy rain) litter pollution monitoring could be implemented in the methodology.

Weaknesses of the method and ways to eliminate them are:

i. When there is a large amount of reed thrown by waves and mixed with debris on the lagoon shore in wrack accumulation zones (wrack lines), it is challenging to make a representative analysis of the presence of micro- and meso-litter. Micro-litter could be stuck on the reed and accidentally discarded while removing the reed from the 1 m^2^ sampling areas. It is proposed to remove and wash the reed layer by layer in a separate bucket on the 1 m^2^ areas (some stems could be cut with a knife) and then drain this water with the resulting suspension through a 2 mm sieve.
ii. Extrapolation of the results from the 2 × 1 m^2^ areas is problematic because it is mainly based on the accumulation zone, while smaller items are missed in the 40 m^2^ rectangular areas.
iii. On the wide shores of lagoons, several wrack lines could be observed. The proposed method should primarily account for the wrack line closest to the water body. The more “distant” wrack lines could be sampled additionally by placing another 40 m^2^ polygon parallel to the first one.
iv. If there are pebbles, gravel, or shells on the lagoon’s shore, it is recommended to use a cascade of 10 mm and 2 mm sieves to remove the increased load on the 2 mm sieve.
v. It is difficult to identify small wet particles and films (black, white, transparent) and distinguish them from objects of biological origin in the field.
vi. While the method implies sampling at quite a small rectangle of 4 × 10 m and thus requires a beach of a least 10 m length, there was a problem finding even such a small sandy coast in some areas. For example, Curonian Lagoon (especially on the eastern and southern coast) has shores covered with reed beds, muddy swamps, or a thick layer of shells of zebra mussels.
vii. In this study, only accessible lagoon/bay beaches with parking spots have been sampled; therefore, rural beaches should be included in the monitoring to understand tourism’s impact better.

## Conclusions

The litter monitoring method developed in this study aims to investigate all-size litter pollution on the beaches of coastal lagoons and bays, which are relatively short, with a larger sediment size fraction than the open-coast beaches. In total, 23 beaches from the inner-coastal waters of the Baltic Sea were investigated using the methodology developed in this study. In two major Baltic Sea lagoons and one bay, 817 litter pieces were found (0.22 pieces/m^2^). Substantial differences were observed in the total number and litter densities between the water bodies. Micro- and meso-litter size category and macro-litter resulted in somewhat equal parts, 339 and 478 pieces, respectively. The latter consisted of various types of construction material (plastic or glass/ceramic), indicating that lagoons could be a potential sink of land-based litter pollution. This study’s findings suggest that litter pieces were mainly introduced to the inner-coastal beaches from the tourism sector, wastewater treatment and stormwater drainage, and land-based industry and trade. We believe that results obtained using a newly established monitoring method are reliable, comparable, and fit the requirements of MSFD.

## Supplementary Information

Supplementary 1

Supplementary 2
